# Supplementary material for: Mating changes the genital microbiome in both sexes of the common bedbug Cimex lectularius across populations
Source: Proc Biol Sci. 2020 Apr 29;287(1926):20200302. doi: 10.1098/rspb.2020.0302 (PMC7282915; doi:10.1098/rspb.2020.0302)
Supplement: Supplementary material [file rspb20200302supp1.pdf]

SUPPLEMENTARY INFORMATION FOR

Mating changes the genital microbiome in both sexes of the common bedbug *Cimex lectularius* across populations

Sara Bellinvia[1], Paul R. Johnston[2], Susan Mbedi[3,4], Oliver Otti[1]

1 Animal Population Ecology, Animal Ecology I, University of Bayreuth, Universitätsstraße 30, 95440 Bayreuth, Germany
2 Institute for Biology, Free University Berlin, Königin-Luise-Straße 1-3, 14195 Berlin, Germany.
3 Museum für Naturkunde - Leibniz-Institute for Evolution and Biodiversity Research, Invalidenstraße 43, 10115 Berlin.
4 Berlin Center for Genomics in Biodiversity Research (BeGenDiv), Königin-Luise-Straße 1-3, 14195 Berlin, Germany.

INDEX:

SUPPLEMENTARY FIGURES

Figure S1 Alpha diversity of virgin and mated bedbugs

Figure S2 PCoA: comparison between cuticular and genital microbiomes

SUPPLEMENTARY TABLES

Table S1 Sample sizes before and after sequencing and quality control

Table S2 Contaminants in controls

Table S6 SVs potentially transmitted from males to females

Table S7 SVs potentially transmitted from females to males

Separate Excel files:

Table S3 Sample information regarding origin, processing date, and order of amplification

Table S4 Read counts for each sample and sequence variant

Table S5 Taxonomic assignment for each sequence variant

SUPPLEMENTARY FIGURES

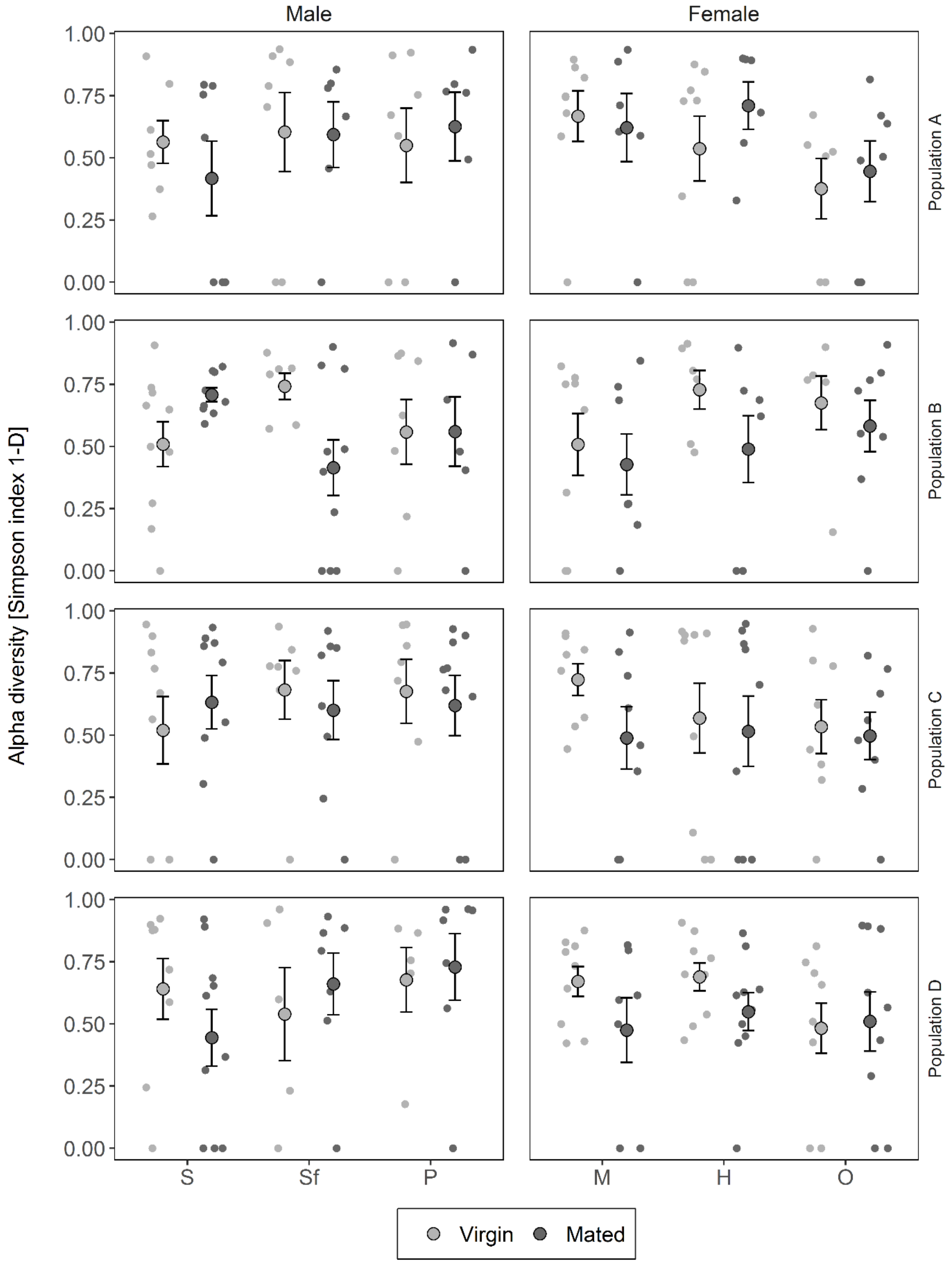

Figure S1 Alpha diversity of each sample in the sperm vesicle (S), the seminal fluid vesicle (Sf), on the paramere (P), in the mesospermalege (M), the haemolymph (H) and the ovary (O). Depicted are means, standard errors of the mean, and all individual data points.

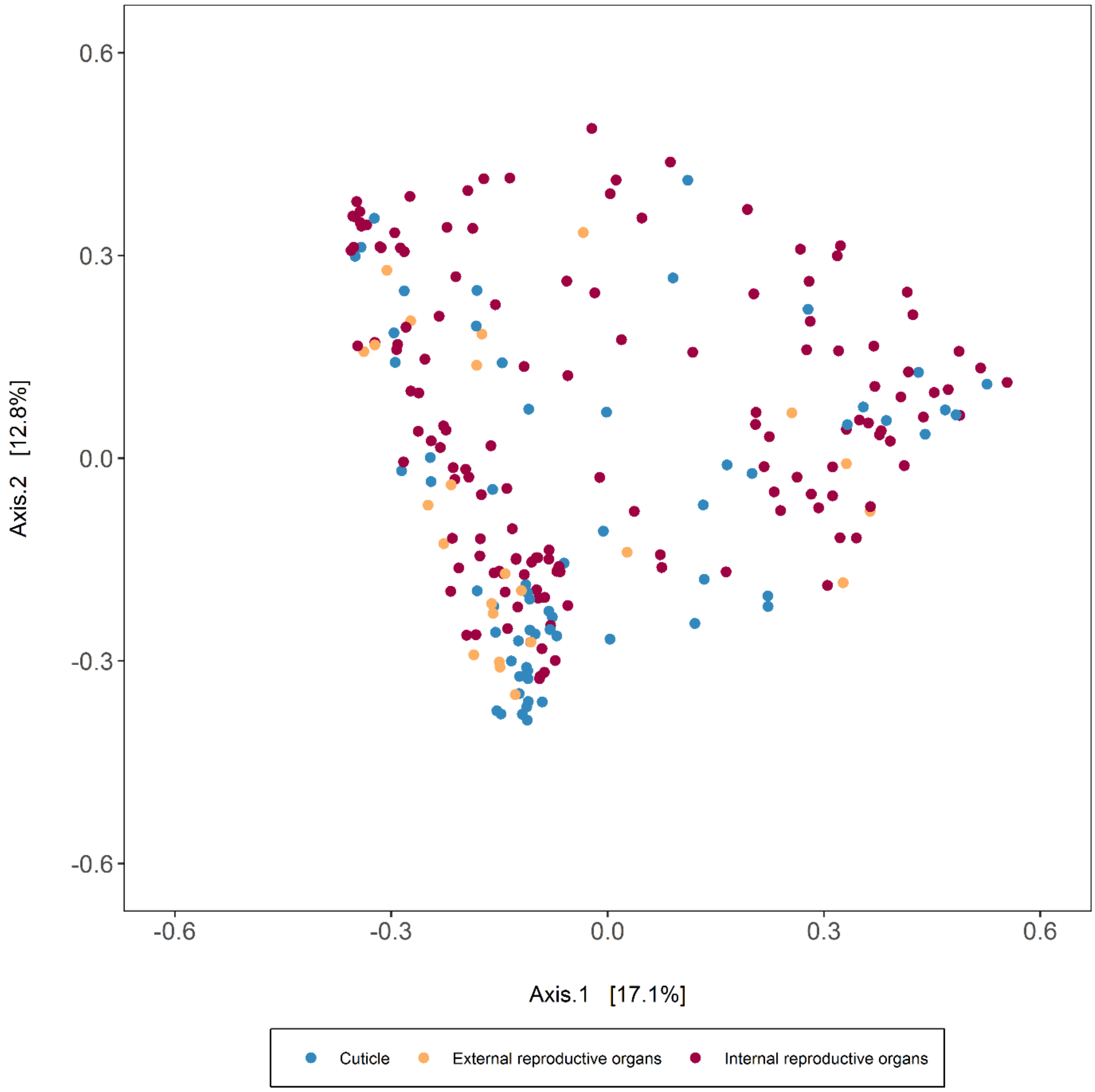

Figure S2 PCoA of microbiomes from cuticle in comparison to the external reproductive organ of males) and internal reproductive organs of both sexes based on Bray-Curtis dissimilarities.

SUPPLEMENTARY TABLES

Table S1 Sample sizes and number of bacterial communities that were successfully sequenced and endured quality filtering for each group of samples. Sampled organs were: cuticle (C), sperm vesicle (S), seminal fluid vesicle (Sf), paramere (P), mesospermalege (M), haemolymph (H) and ovary (O).

Population	Sex	Organ	Mating status	N total	N sequenced
A	Male	C	Virgin	10	7
A	Male	C	Mated	10	6
A	Male	S	Virgin	10	7
A	Male	S	Mated	11	7
A	Male	Sf	Virgin	10	7
A	Male	Sf	Mated	10	6
A	Male	P	Virgin	10	7
A	Male	P	Mated	10	6
A	Female	C	Virgin	11	7
A	Female	C	Mated	11	8
A	Female	M	Virgin	10	8
A	Female	M	Mated	10	6
A	Female	H	Virgin	11	8
A	Female	H	Mated	10	6
A	Female	O	Virgin	10	6
A	Female	O	Mated	9	7
B	Male	C	Virgin	10	9
B	Male	C	Mated	10	8
B	Male	S	Virgin	10	10
B	Male	S	Mated	10	9
B	Male	Sf	Virgin	10	6
B	Male	Sf	Mated	10	10
B	Male	P	Virgin	12	7
B	Male	P	Mated	8	6
B	Female	C	Virgin	10	10
B	Female	C	Mated	10	8
B	Female	M	Virgin	10	8
B	Female	M	Mated	10	7
B	Female	H	Virgin	10	6
B	Female	H	Mated	10	8
B	Female	O	Virgin	10	6
B	Female	O	Mated	10	8
C	Male	C	Virgin	10	9
C	Male	C	Mated	10	7
C	Male	S	Virgin	10	9
C	Male	S	Mated	10	9
C	Male	Sf	Virgin	10	7
C	Male	Sf	Mated	10	8
C	Male	P	Virgin	10	7
C	Male	P	Mated	10	9
C	Female	C	Virgin	10	9

C	Female	C	Mated	10	8
C	Female	M	Virgin	10	8
C	Female	M	Mated	10	8
C	Female	H	Virgin	10	9
C	Female	H	Mated	10	9
C	Female	O	Virgin	10	8
C	Female	O	Mated	10	8
D	Male	C	Virgin	10	8
D	Male	C	Mated	10	8
D	Male	S	Virgin	10	8
D	Male	S	Mated	10	10
D	Male	Sf	Virgin	10	5
D	Male	Sf	Mated	10	7
D	Male	P	Virgin	10	5
D	Male	P	Mated	10	7
D	Female	C	Virgin	10	8
D	Female	C	Mated	10	10
D	Female	M	Virgin	10	9
D	Female	M	Mated	10	7
D	Female	H	Virgin	10	9
D	Female	H	Mated	10	10
D	Female	O	Virgin	10	9
D	Female	O	Mated	10	9

Table S2 Contaminants found with the *decontam* package (Davis et al. 2018) based on prevalences in controls. Given are the contaminants and their prevalences within each type of control, i.e. control for dissection, control for DNA isolation, control for target PCR, and control for indexing PCR.

Contaminant	Dissection (N=1)	DNA isolation (N=1)	Target PCR (N=16)	Indexing PCR (N=13)
Unclassified Enterobacteriaceae	1	0	0.81	0.62
Wolbachia sp.	0	0	0.75	0.77
Bradyrhizobium sp.	0	0	0.44	0.08
Stenotrophomonas sp.	0	0	0.25	0.08
Myroides sp.	0	0	0.13	0.00
Methylobacterium sp.	0	0	0.38	0.08
Pseudomonas sp.	0	0	0.13	0.00
Methylobacterium sp.	0	0	0.19	0.08
*Enterococcus*sp.	0	0	0.13	0.00
Sphingomonas sp.	0	0	0.25	0.00
Unclassified Streptophyta	0	0	0.13	0.00
Curvibacter sp.	0	0	0.25	0.08
Rickettsia sp.	0	0	0.06	0.08
Sphingomonas sp.	0	0	0.19	0.00
Curvibacter sp.	0	0	0.19	0.00
Unclassified Streptophyta	0	0	0.06	0.00
Unclassified Erythrobacteraceae	0	0	0.13	0.08
Janthinobacterium sp.	0	0	0.13	0.00
Unclassified Erythrobacteraceae	0	0	0.13	0.00
Unclassified Lactobacillales	0	0	0.06	0.00
Janthinobacterium sp.	0	0	0.13	0.00
Pseudomonas sp.	0	0	0.06	0.08
Unclassified Lactobacillales	0	0	0.06	0.00
Sphingomonas sp.	0	0	0.13	0.00
Methylobacterium sp.	0	0	0.06	0.00
Methylobacterium sp.	0	0	0.06	0.00
Novosphingobium sp.	0	0	0.13	0.08
Paracoccus sp.	0	0	0.13	0.00
Renibacterium sp.	0	0	0.13	0.00
Unclassified Pseudomonadaceae	0	0	0.06	0.00
Unclassified Rhodospirillaceae	0	0	0.06	0.00
Bacillus thermoalkalophilus	0	0	0.06	0.00
Prevotella sp.	0	0	0.13	0.00
Pseudoxanthomonas taiwanensis	0	0	0.13	0.00
Unclassified Rhodospirillaceae	0	0	0.06	0.00
Rickettsia sp.	0	0	0.06	0.08
Unclassified Pseudomonadaceae	0	0	0.06	0.00
Thermomonas sp.	0	0	0.06	0.00
Unclassified Erythrobacteraceae	0	0	0.06	0.00
Unclassified Rhodospirillaceae	0	0	0.06	0.00
Bacillus thermoalkalophilus	0	0	0.06	0.00
Thermus sp.	0	0	0.06	0.00

Rubrivivax sp.	0	0	0.06	0.00
Bosea genosp.	0	0	0.06	0.00
Unclassified Pseudomonadaceae	0	0	0.06	0.00
Unclassified Phyllobacteriaceae	0	0	0.06	0.00
Sphingobacterium sp.	0	0	0.06	0.00
Unclassified Comamonadaceae	0	0	0.06	0.00
Marinobacter sp.	0	0	0.06	0.00
Bosea genosp.	0	0	0.06	0.00
Marinobacter sp.	0	0	0.06	0.00
Nevskia sp.	0	0	0.06	0.00

Table S6 SVs potentially transmitted from males to females. Given are prevalence and relative abundance of all SVs that were found in mated females and in virgin males but not in virgin females.

ID	SV	Prevalence	Min. relative abundance	Max. relative abundance
Population A		*(N=19)*		
SV30	Unclassified Aeromonas	6	0.0009	0.0065
SV44	*Staphylococcus* sp.	2	0.0001	0.0003
SV51	*Bradyrhizobium* sp.	6	0.0006	0.0091
SV66	*Caulobacter* sp.	7	0.0001	0.0045
SV68	*Alcanivorax* sp.	4	0.0004	0.0024
SV69	*Brevibacterium* sp.	1	0.0004	0.0004
SV73	*Staphylococcus* sp.	2	0.0010	0.0018
SV77	*Alcanivorax* sp.	4	0.0003	0.0017
SV83	*Staphylococcus* sp.	1	0.0010	0.0010
SV86	Unclassified Comamonadaceae	3	0.0004	0.0020
SV94	*Bradyrhizobium* sp.	3	0.0003	0.0020
SV97	*Cutibacterium acnes*	5	0.0001	0.0029
SV98	*Acinetobacter* sp.	2	0.0003	0.0016
SV99	*Bradyrhizobium* sp.	4	0.0005	0.0009
SV100	Unclassified Gammaproteobacteria	1	0.0002	0.0002
SV106	*Corynebacterium* sp.	5	0.0002	0.0023
SV109	*Paracoccus* sp.	1	0.0014	0.0014
SV111	*Pseudomonas* sp.	3	0.0005	0.0008
SV114	*Cloacibacterium* sp.	2	0.0000	0.0007
SV115	Unclassified Alcaligenaceae	1	0.0003	0.0003
SV116	*Acinetobacter* sp.	5	0.0001	0.0018
SV117	Unclassified Lactobacillaceae	1	0.0004	0.0004
SV118	*Cloacibacterium* sp.	2	0.0002	0.0005
SV120	*Lactobacillus* sp.	1	0.0002	0.0002
SV122	Unclassified Gammaproteobacteria	2	0.0003	0.0018
SV124	*Pseudomonas* sp.	2	0.0006	0.0006
SV125	*Acinetobacter lwoffii*	4	0.0003	0.0021
SV126	*Comamonas* sp.	3	0.0004	0.0015
SV135	Unclassified Comamonadaceae	2	0.0004	0.0047
Population B		*(N=22)*		
SV135	Unclassified Comamonadaceae	1	0.0030	0.0030
Population C		*(N=25)*		
SV111	*Pseudomonas* sp.	4	0.0002	0.0063
SV124	*Pseudomonas* sp.	1	0.0004	0.0004
SV140	Unclassified Rhodobacteraceae	1	0.0001	0.0001
Population D		*(N=26)*		
SV33	*Stenotrophomonas* sp.	1	0.0003	0.0003
SV117	Unclassified Lactobacillaceae	3	0.0001	0.0023
SV118	*Cloacibacterium* sp.	2	0.0001	0.0009

SV138	*Corynebacterium* sp.	1	0.0003	0.0003

Table S7 SVs potentially transmitted from females to males. Given are prevalence and relative abundance of all SVs that were found in mated males and in virgin females but not in virgin males.

ID	SV	Prevalence	Min. relative abundance	Max. relative abundance
Population A			*(N=19)*	
-	-	-	-	-
Population B			*(N=25)*	
SV90	*Bradyrhizobium* sp.	2	0.0011	0.0033
SV93	*Halomonas* sp.	2	0.0015	0.0024
SV122	Unclassified Gammaproteobacteria	2	0.0004	0.0008
SV134	Unclassified Gammaproteobacteria	2	0.0004	0.0009
Population C			*(N=26)*	
-	-	-	-	-
Population D			*(N=24)*	
SV44	*Staphylococcus* sp.	1	0.0016	0.0016
SV108	*Lactobacillus* sp.	1	0.0002	0.0002

SUPPLEMENTARY REFERENCES

Davis NM, Proctor DM, Holmes SP, Relman DA, Callahan BJ. 2018 Simple statistical identification and removal of contaminant sequences in marker-gene and metagenomics data. *Microbiome* **6**, 226. (doi:10.1186/s40168-018-0605-2)
